# Synthesis and structure of a 1:1 cocrystal of *N*,*N*′-bis­(pyridin-3-ylmeth­yl)pyromellitic di­imide and naphthalene-2,6-di­carb­oxy­lic acid

**DOI:** 10.1107/S2056989026006110

**Published:** 2026-06-23

**Authors:** Luccile Mbonzhe, Eustina Batisai

**Affiliations:** a University of Venda, P Bag X5050, Thohoyandou, 0950, South Africa; University of Aberdeen, United Kingdom

**Keywords:** cocrystal, pyromellitic di­imide, Hirshfeld surface analysis, naphthalenedi­carb­oxy­lic acid

## Abstract

In the title cocrystal, C_22_H_14_N_4_O_4_·C_12_H_8_O_4_, both components are completed by crystallographic inversion symmetry and the dihedral angle between the central fused ring system and the pendant pyridine ring is 66.21 (5)°. In the extended structure, the components are linked by O—H⋯N hydrogen bonds, generating [001] chains, and the packing is consolidated by π–π stacking and C—H⋯O inter­actions.

## Abstract 2

The title cocrystal features infinite chains of alternating components linked by O—H⋯N hydrogen bonds.

## 1. Chemical context

*N*,*N*′-Bis(pyridin-3-ylmeth­yl)pyromellitic di­imide, C_22_H_14_N_4_O_4_, (Lig1) consists of a pyromellitic di­imide core linked to *meta*-substituted pyridyl groups *via* –CH_2_– linkages. These rotatable linkages impart conformational flexibility, allowing Lig1 to adopt *Z_C-_* and *Z_T-_* modes, where *Z* denotes an *anti* orientation of the pyridyl rings, while *C* (*cis*) and *T* (*trans*) describe the relative positions of the pyridyl nitro­gen atoms (Yan *et al.*, 2010 ▸). The pyromellitic di­imide core further promotes π–π stacking inter­actions, contributing to supra­molecular assembly. Owing to its semi-rigid nature, Lig1 has been widely employed in the construction of metal–organic frameworks (MOFs) exhibiting diverse topologies, with potential applications in gas sorption (Li *et al.*, 2012 ▸) and fluorescence (Huang *et al.*, 2020 ▸; Li *et al.*, 2018 ▸).

Deprotonated naphthalene di­carb­oxy­lic acid (C_12_H_8_O_4_; NDC) ligands are also widely used to prepare MOFs with potential applications in gas storage, gas separation, luminescence and catalysis (Gangu *et al.*, 2017 ▸). Their aromatic ring system allows for increased π–π stacking inter­actions, enhancing supra­molecular recognition and enabling the formation of complex polymer networks.
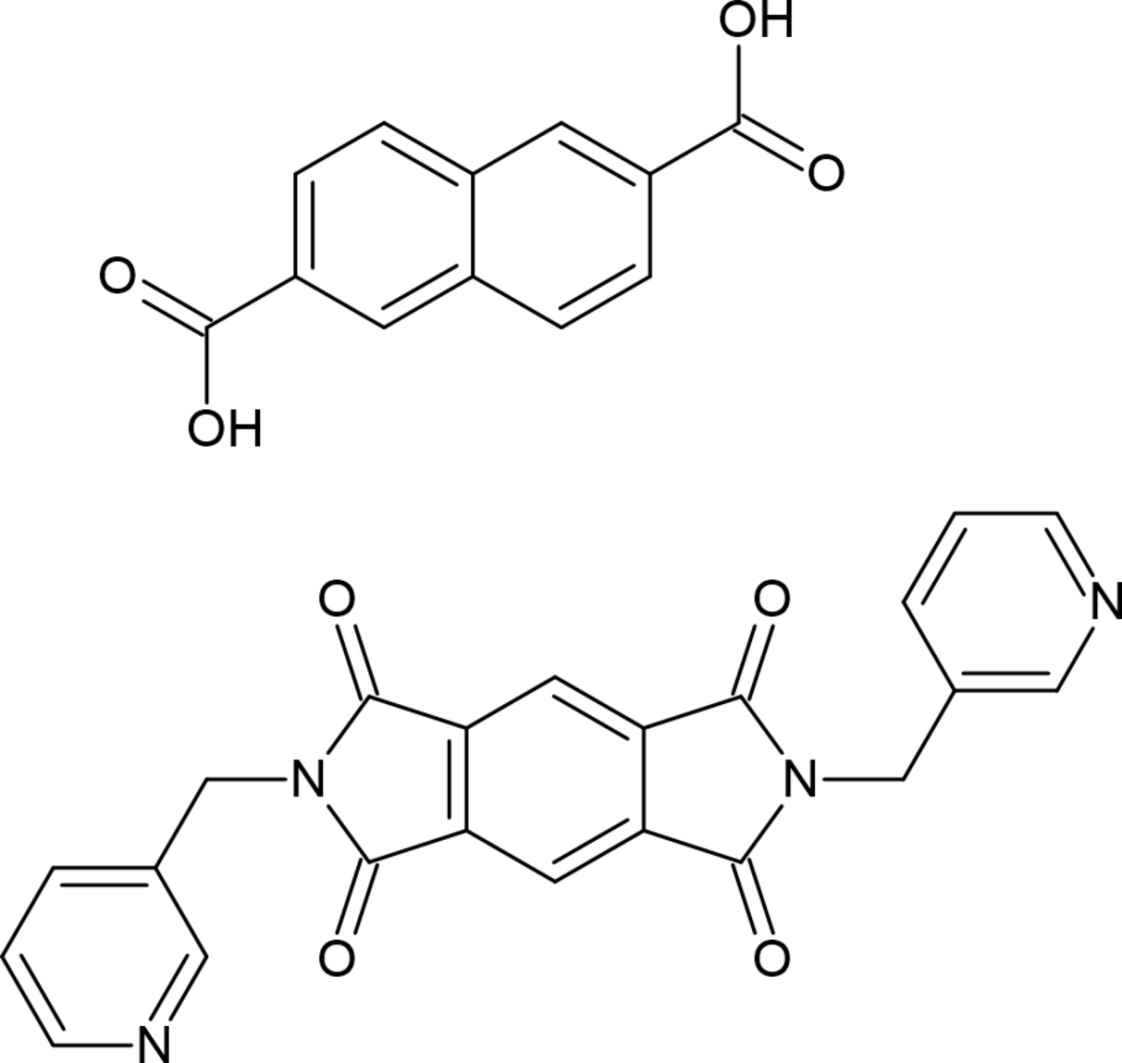


The aim of this work was to prepare a zinc mixed-ligand MOF containing Lig1 and NDC. However, single-crystal X-ray diffraction (SCXRD) revealed that a 1:1 cocrystal of Lig1 and NDC, (**I**), had formed from the solvothermal reaction and we now describe its structure.

## 2. Structural commentary

**Figure 1:**
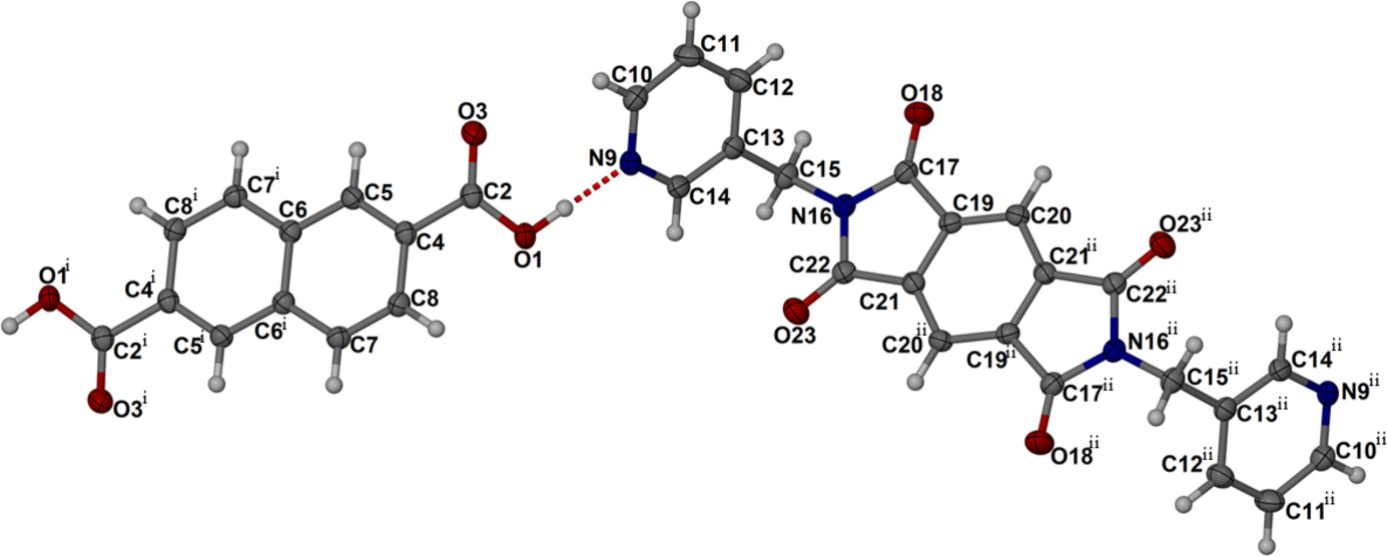
The mol­ecular structure of (**I**) with displacement ellipsoids drawn at the 70% probability level. [Symmetry codes: (i) 2 − *x*, 1 − *y*, 2 − *z*; (ii) 1 − *x*, 1 − *y*, −*z*].

Compound (**I**) crystallizes in the triclinic space group *P*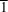 with half a mol­ecule of Lig1 and half a mol­ecule of NDC in the asymmetric unit (Fig. 1 ▸). Both complete mol­ecules are generated by crystallographic inversion centres at (1/2, 1/2, 0) and (1, 1/2, 1) for the asymmetric atoms of Lig1 and NDC, respectively. The Lig1 mol­ecule adopts a *Z_T-_* mode and the dihedral angle between the central fused ring system and the pendant pyridine ring is 66.21 (5)°.

## 3. Supra­molecular features

**Figure 2:**
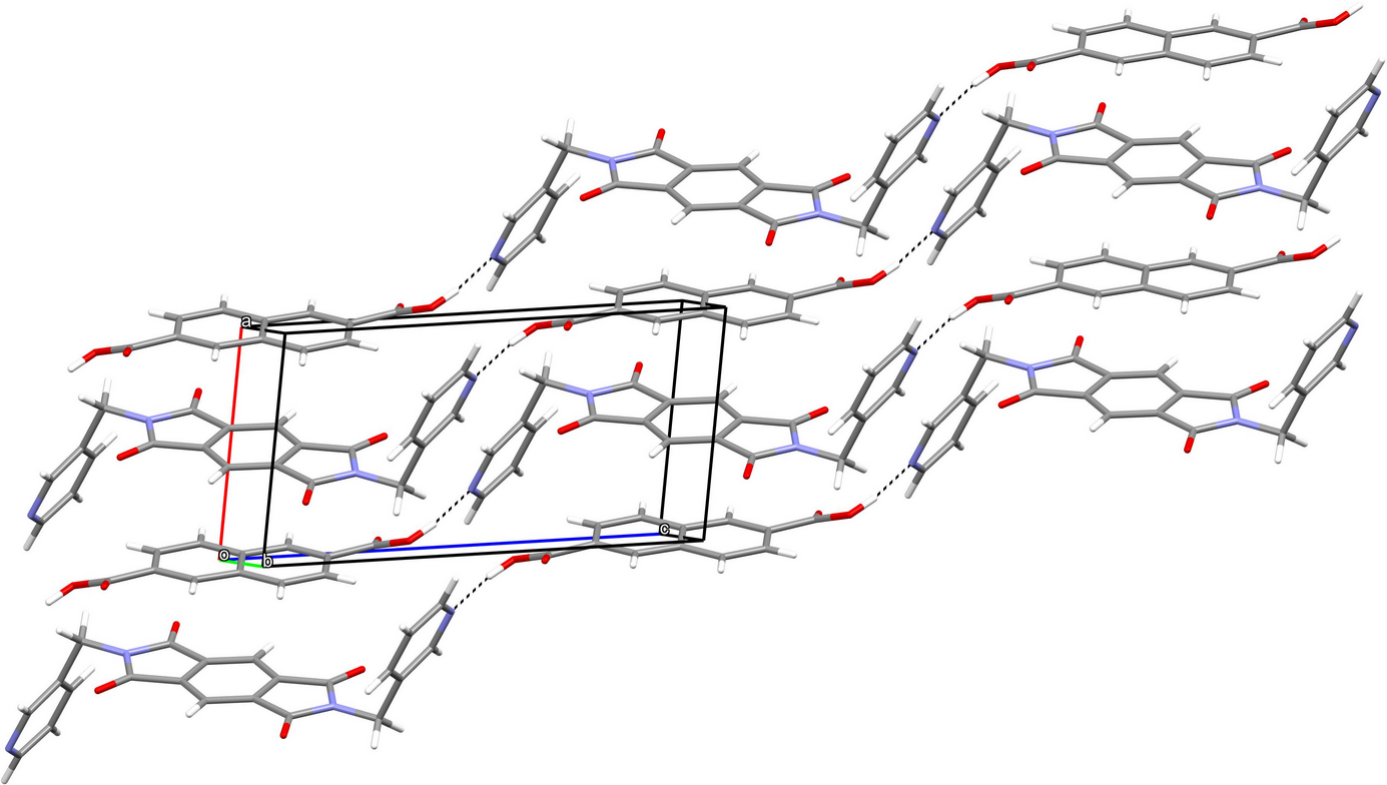
The packing of (**I**) viewed down the *b*-axis direction.

In the extended structure of (**I**), the NDC mol­ecule links to Lig1 *via* an O1—H1⋯N9 hydrogen bond between the carb­oxy­lic acid group of NDC and the py-*N* of Lig1, and a secondary inter­action between the Ar-H atom of Lig 1 and the C=O group of the NDC mol­ecule (C10—H10⋯O3). These inter­actions result in extended chains of alternating Lig1 and NDC mol­ecules running along the crystallographic *c-*axis direction (Table 1 ▸, Fig. 2 ▸).

The chains stack in the *ac* plane in an offset arrangement facilitated by π–π stacking inter­actions [centroid-to-centroid distance = 3.767 (1) Å] between the pyridyl moieties of Lig1 of adjacent chains, as well as π–π inter­actions [centroid-to-centroid distance = 3.761 (1) Å] between the pyromellitic moiety of Lig1 and the naphthalene moiety of NDC of adjacent chains. Additionally, the chains inter­act with neighboring chains *via* C—H⋯O inter­actions in the *ac* plane (C15—H15*A*⋯O3) as well as in the *b*-axis direction (C11—H11⋯O23) (Fig. 2 ▸).

## 4. Hirshfeld surface analysis

**Figure 3:**
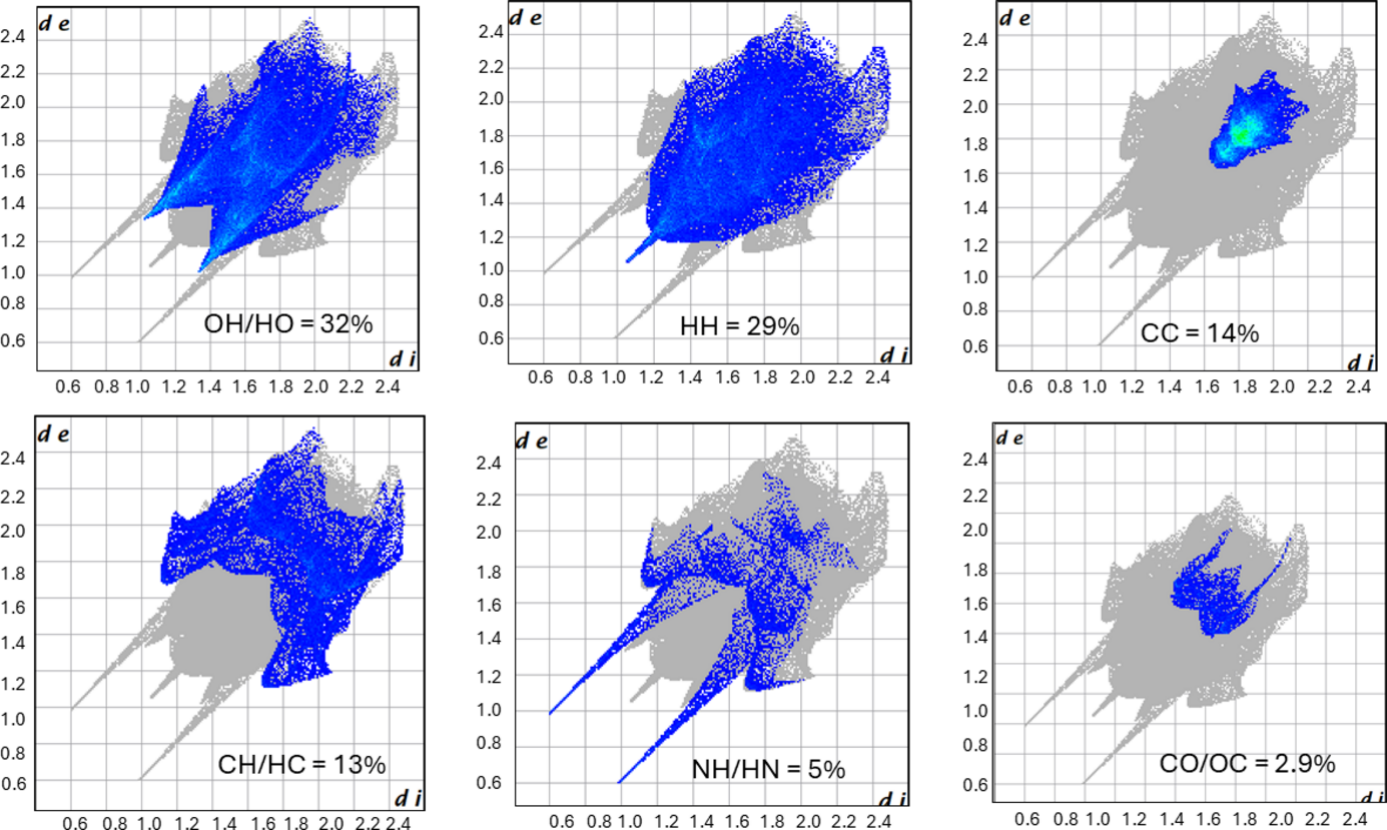
Two-dimensional fingerprint plots for (**I**) showing the various contributions to the Hirshfeld surface.

To further qu­antify the nature and relative contributions of inter­molecular inter­actions within the crystal structure of (**I**), two-dimensional fingerprint plots were generated using *CrystalExplorer* (Spackman *et al.* 2021 ▸). The Hirshfeld surface was constructed from a hydrogen-bonded unit comprising one NDC and one Lig1 mol­ecule. The breakdown of inter­molecular contacts and their percentage contributions to the total Hirshfeld surface are presented in Fig. 3 ▸. The contributions follow the order O⋯H/H⋯O (32%) > H⋯H (29%) > C⋯C (14%) > C⋯H/H⋯C (13%) > N⋯H/H⋯N (5%) > C⋯O/O⋯C (2.9%). Although O⋯H/H⋯O inter­actions make the largest contribution to the Hirshfeld surface, these contacts are relatively long, with the closest atom–atom distance of approximately 2.3 Å, and are mainly associated with weaker C—H⋯O inter­actions. H⋯H contacts also contribute significantly, indicating that van der Waals inter­actions play an important role in consolidating the crystal structure. The C⋯C and C⋯H inter­actions contribute 14% and 13%, respectively, and can be attributed to π–π stacking and C-H⋯π inter­actions. N⋯H/H⋯N inter­actions, associated with O—H⋯N hydrogen bonding, contribute a smaller proportion (5%) but represent the shortest inter­molecular contacts, with a closest atom–atom distance of approximately 1.6 Å.

## 5. Thermogravimetric analysis (TGA) and powder X-ray diffraction (PXRD)

**Figure 4:**
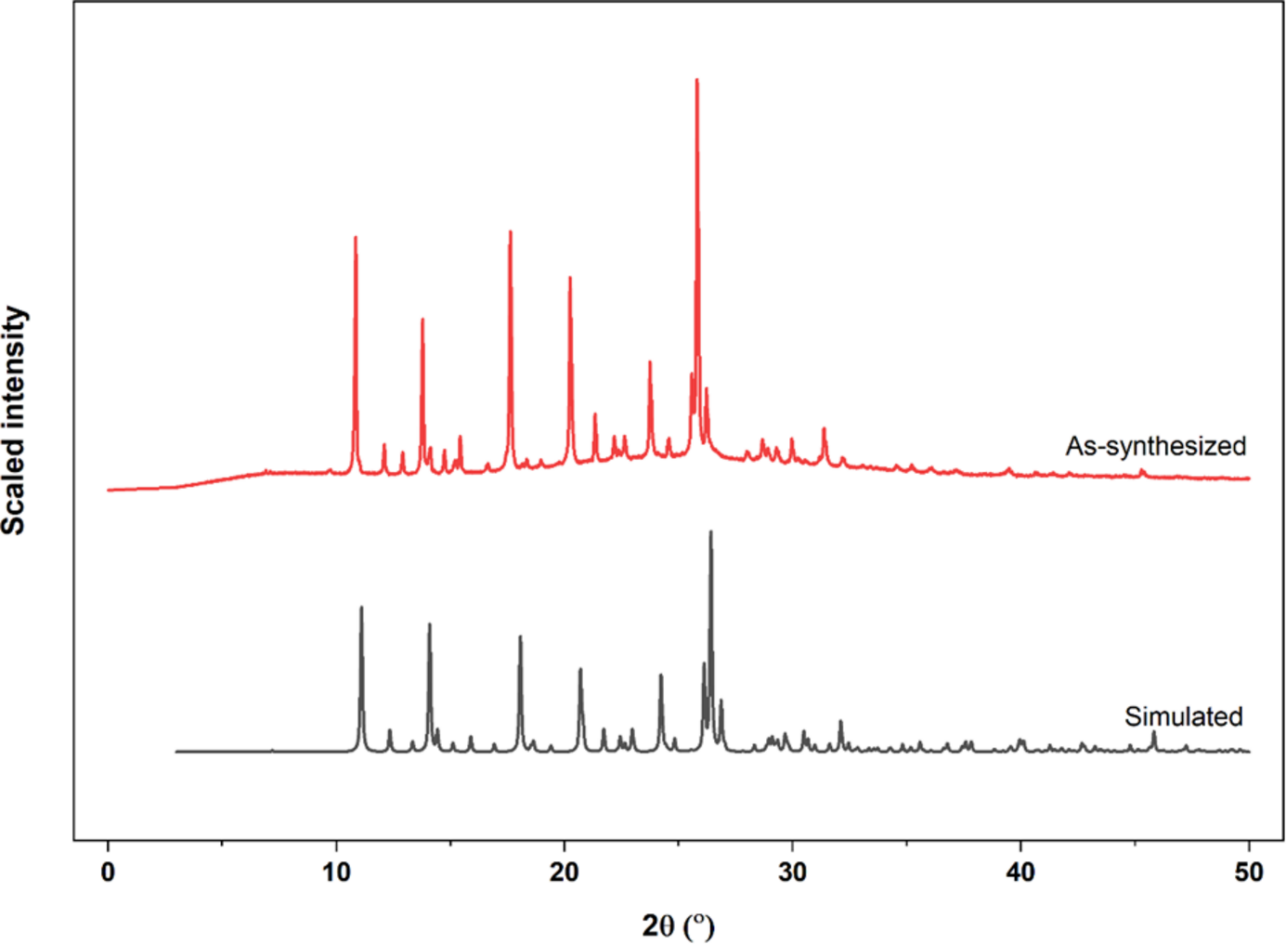
Experimental PXRD pattern of (**I**) (red) overlaid with the simulated pattern generated from SCXRD data (black), confirming phase purity.

**Figure 5:**
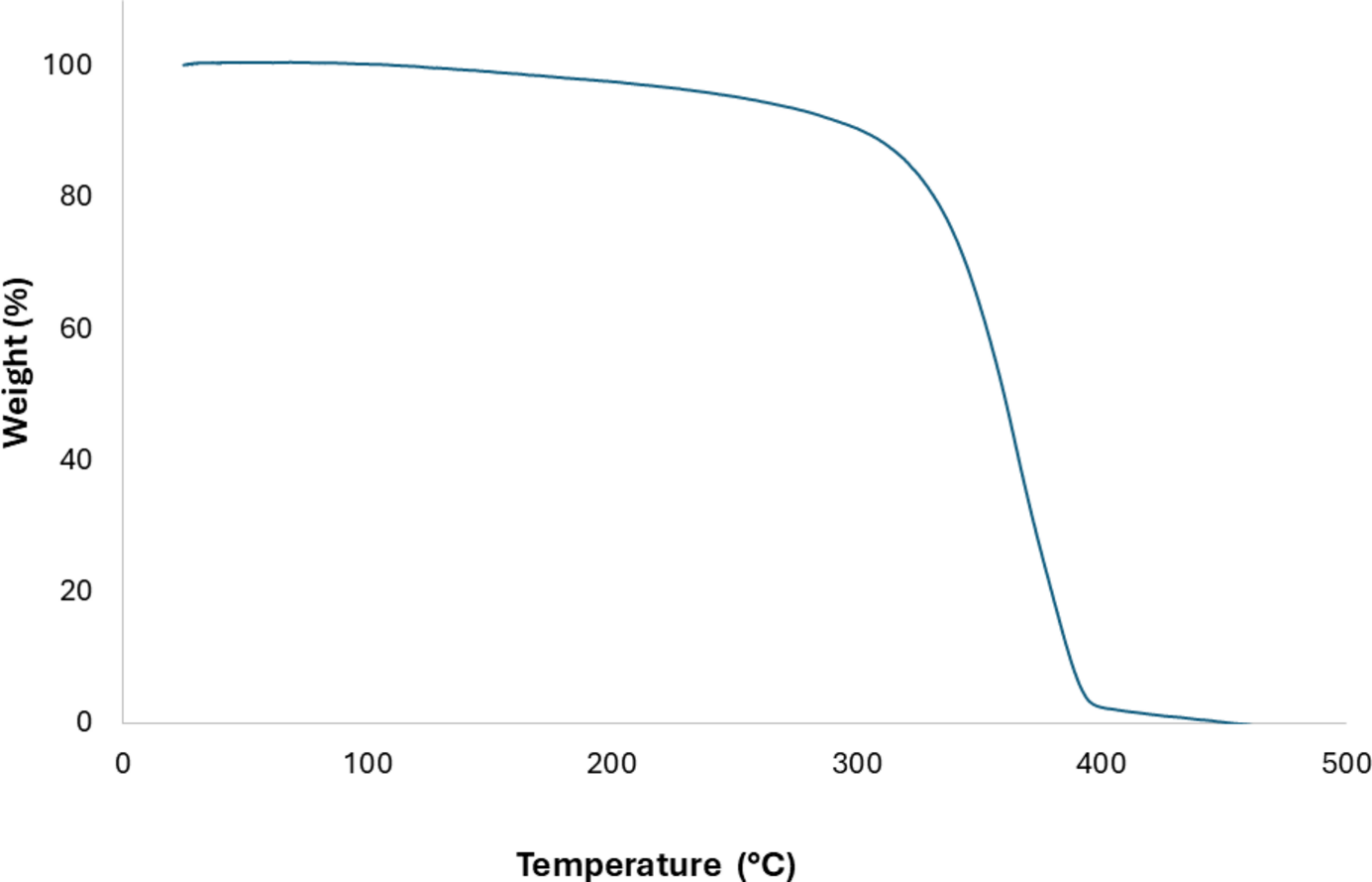
TGA curve of (**I**). Decomposition starts at 290 °C.

PXRD analysis was performed to assess the bulk phase purity of (**I**). A comparison of the PXRD pattern of the as-synthesized sample with the simulated pattern of (**I**) shows good agreement, which indicates phase purity of the sample (Fig. 4 ▸). The TGA curve shows that the cocrystal decomposes at 290** °**C (Fig. 5 ▸).

## 6. Database survey

A search of the Cambridge Structural Database (CSD) (Groom *et al.*, 2016 ▸) showed that Lig1 has been used mostly in the preparation of coordination complexes. Of the 20 crystal structures deposited Co [DIBBAU, DIBBEY, DIBBIC (Li *et al.*, 2018 ▸), OWEYEV (Li *et al.*, 2011 ▸)], Zn [OWEYUL (Li *et al.*, 2011 ▸), FISCEQ (Lü *et al.* 2005*b* ▸), PALYIL (Lü *et al.* 2005*a* ▸)], Cd [FISCAM (Lü *et al.* 2005*b* ▸), PADHOT (Chai *et al.* 2010 ▸), PALYUX (Lü *et al.* 2005*a* ▸), ZAVQIZ (Li *et al.*, 2017 ▸), ZAXSOI (Li *et al.*, 2012 ▸)], Ag (QAHBEI, QAHBIM; Yan *et al.*, 2011 ▸), Hg [HUHZUI (Huang *et al.*, 2020 ▸), PEVYUL (Li *et al.*, 2007 ▸)], Ni (HUJBAS; Huang *et al.*, 2020 ▸) and Mn [OWEXOE (Li *et al.*, 2011 ▸), ZAVQEV Li *et al.*, 2017 ▸)] and one entry is a salt of protonated Lig1 and a perchlorate ion (FISBUF; Lü *et al.*, 2005 ▸ ▸). The coordination complexes exhibit structural diversity, with some investigated for CO_2_ sorption (Li *et al.*, 2012 ▸) and fluorescence properties (Huang *et al.*, 2020 ▸; Li *et al.*, 2018 ▸). A search on the CSD for NDC produced 683 coordination complexes containing NDC either by itself or in combination with other ligands. Additionally, there are 40 hits where NDC features in cocrystals or salts.

## 7. Synthesis and crystallization

Lig1 was synthesized according to a reported procedure (Li *et al.* 2009 ▸). Compound (**I**) crystallized from a solvothermal reaction of Lig1 (10 mg, 0.025 mmol), NDC (16 mg, 0.074 mmol), and Zn(NO_3_)_2_·6H_2_O (20 mg, 0.067 mmol) in 3 ml of *N*,*N*-di­methyl­formamide (DMF) at 373 K. Cream crystals of (**I**) formed after 3 days. The vial was then removed from the oven and washed with 2 ml of DMF.

## 8. Refinement

Crystal data, data collection and structure refinement details are summarized in Table 2 ▸. C-bound H atoms were positioned (C—H = 0.95–0.99 Å) geometrically and refined as riding *U*_iso_(H) = 1.2*U*_eq_(C). The OH H atom was found in a difference map and refined with *U*_iso_(H) = 1.5*U*_eq_(O).

## Supplementary Material

Crystal structure: contains datablock(s) I. DOI: 10.1107/S2056989026006110/hb8224sup1.cif

Structure factors: contains datablock(s) I. DOI: 10.1107/S2056989026006110/hb8224Isup2.hkl

Supporting information file. DOI: 10.1107/S2056989026006110/hb8224Isup3.mol

CCDC reference: 2561484

Additional supporting information:  crystallographic information; 3D view; checkCIF report

## Figures and Tables

**Table 1:** Hydrogen-bond geometry (Å, °)

*D*—H⋯*A*	*D*—H	H⋯*A*	*D*⋯*A*	*D*—H⋯*A*
O1—H1⋯N9	0.95 (1)	1.64 (1)	2.5806 (13)	172 (2)
C15—H15*A*⋯O3^i^	0.99	2.56	3.4073 (14)	143
C11—H11⋯O23^ii^	0.95	2.51	3.2402 (15)	134
C10—H10⋯O3^iii^	0.95	2.64	3.2414 (15)	122
C11—H11⋯O3^iii^	0.95	2.62	3.2207 (15)	122

Symmetry codes: (i) 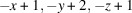; (ii) 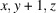; (iii) 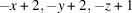.

**Table 2:** Experimental details

Crystal data	
Chemical formula	C_22_H_14_N_4_O_4_·C_12_H_8_O_4_
*M*_r_	614.55
Crystal system, space group	Triclinic, *P* 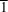
Temperature (K)	130
*a*, *b*, *c* (Å)	6.9314 (2), 8.3554 (3), 12.5463 (4)
α, β, γ (°)	79.716 (1), 80.977 (1), 74.432 (1)
*V* (Å^3^)	684.15 (4)
*Z*	1
Radiation type	Mo *K*α
μ (mm^−1^)	0.11
Crystal size (mm)	0.28 × 0.17 × 0.13

Data collection	
Diffractometer	BRUKER D8 QUEST
Absorption correction	Multi-scan (*SADABS*; Krause *et al.*, 2015 ▸)
*T*_min_, *T*_max_	0.650, 0.746
No. of measured, independent and observed [*I* > 2σ(*I*)] reflections	23721, 3391, 3048
*R*_int_	0.040
(sin θ/λ)_max_ (Å^−1^)	0.667

Refinement	
*R*[*F*^2^ > 2σ(*F*^2^)], *wR*(*F*^2^), *S*	0.039, 0.107, 1.10
No. of reflections	3391
No. of parameters	211
No. of restraints	1
H-atom treatment	H atoms treated by a mixture of independent and constrained refinement
Δρ_max_, Δρ_min_ (e Å^−3^)	0.36, −0.28

Computer programs: *APEX4* and *SAINT* (Bruker, 2022 ▸), *SHELXT* (Sheldrick, 2015*a* ▸), *SHELXL2019/3* (Sheldrick, 2015*b* ▸) and *X-SEED* (Barbour, 2020 ▸).

## supplementary crystallographic information

### N,N'-Bis(pyridin-3-ylmethyl)pyromellitic diimide–naphthalene-2,6-dicarboxylic acid (1/1) Crystal data

**Table:**

C_22_H_14_N_4_O_4_·C_12_H_8_O_4_	*Z* = 1
*M**_r_* = 614.55	*F*(000) = 318
Triclinic, *P*1	*D*_x_ = 1.492 Mg m^−^^3^
*a* = 6.9314 (2) Å	Mo *K*α radiation, λ = 0.71073 Å
*b* = 8.3554 (3) Å	Cell parameters from 9965 reflections
*c* = 12.5463 (4) Å	θ = 2.6–28.3°
α = 79.716 (1)°	µ = 0.11 mm^−^^1^
β = 80.977 (1)°	*T* = 130 K
γ = 74.432 (1)°	Block, yellow
*V* = 684.15 (4) Å^3^	0.28 × 0.17 × 0.13 mm

### N,N'-Bis(pyridin-3-ylmethyl)pyromellitic diimide–naphthalene-2,6-dicarboxylic acid (1/1) Data collection

**Table:**

BRUKER D8 QUEST diffractometer	3391 independent reflections
Radiation source: sealed tube	3048 reflections with *I* > 2σ(*I*)
Detector resolution: 7.39 pixels mm^-1^	*R*_int_ = 0.040
ω scans	θ_max_ = 28.3°, θ_min_ = 2.6°
Absorption correction: multi-scan (SADABS; Krause *et al.*, 2015)	*h* = −9→8
*T*_min_ = 0.650, *T*_max_ = 0.746	*k* = −11→11
23721 measured reflections	*l* = −16→16

### N,N'-Bis(pyridin-3-ylmethyl)pyromellitic diimide–naphthalene-2,6-dicarboxylic acid (1/1) Refinement

**Table:**

Refinement on *F*^2^	Primary atom site location: dual
Least-squares matrix: full	Hydrogen site location: mixed
*R*[*F*^2^ > 2σ(*F*^2^)] = 0.039	H atoms treated by a mixture of independent and constrained refinement
*wR*(*F*^2^) = 0.107	*w* = 1/[σ^2^(*F*_o_^2^) + (0.0491*P*)^2^ + 0.3031*P*] where *P* = (*F*_o_^2^ + 2*F*_c_^2^)/3
*S* = 1.10	(Δ/σ)_max_ < 0.001
3391 reflections	Δρ_max_ = 0.36 e Å^−^^3^
211 parameters	Δρ_min_ = −0.28 e Å^−^^3^
1 restraint	

### N,N'-Bis(pyridin-3-ylmethyl)pyromellitic diimide–naphthalene-2,6-dicarboxylic acid (1/1) Special details

**Table:**

Geometry. All esds (except the esd in the dihedral angle between two l.s. planes) are estimated using the full covariance matrix. The cell esds are taken into account individually in the estimation of esds in distances, angles and torsion angles; correlations between esds in cell parameters are only used when they are defined by crystal symmetry. An approximate (isotropic) treatment of cell esds is used for estimating esds involving l.s. planes.

### N,N'-Bis(pyridin-3-ylmethyl)pyromellitic diimide–naphthalene-2,6-dicarboxylic acid (1/1) Fractional atomic coordinates and isotropic or equivalent isotropic displacement parameters (Å^2^)

**Table:**

	*x*	*y*	*z*	*U*_iso_*/*U*_eq_	
O1	0.94933 (14)	0.56834 (11)	0.61477 (7)	0.0239 (2)	
H1	0.887 (2)	0.6401 (17)	0.5555 (9)	0.036*	
O18	0.26446 (15)	0.92395 (11)	0.09152 (7)	0.0272 (2)	
O23	0.50906 (14)	0.42909 (11)	0.30621 (7)	0.0243 (2)	
O3	0.94674 (14)	0.81605 (11)	0.66050 (7)	0.0233 (2)	
N9	0.74937 (16)	0.76234 (12)	0.46376 (8)	0.0198 (2)	
N16	0.36780 (14)	0.69532 (12)	0.22228 (8)	0.0174 (2)	
C7	1.08872 (17)	0.31773 (14)	0.92125 (9)	0.0174 (2)	
H7	1.139624	0.199353	0.934264	0.021*	
C8	1.06660 (16)	0.39725 (14)	0.81661 (9)	0.0171 (2)	
H8	1.098256	0.333481	0.757836	0.021*	
C4	0.99645 (16)	0.57470 (14)	0.79612 (9)	0.0161 (2)	
C2	0.96390 (16)	0.66531 (14)	0.68329 (9)	0.0175 (2)	
C14	0.59674 (18)	0.72163 (14)	0.43038 (9)	0.0199 (2)	
H14	0.570054	0.615645	0.459367	0.024*	
C13	0.47578 (17)	0.82771 (14)	0.35519 (9)	0.0170 (2)	
C15	0.30468 (17)	0.77764 (14)	0.31994 (9)	0.0188 (2)	
H15B	0.252472	0.700467	0.379958	0.023*	
H15A	0.193824	0.878866	0.305142	0.023*	
C17	0.33909 (17)	0.77633 (14)	0.11648 (9)	0.0182 (2)	
C19	0.41928 (16)	0.64345 (13)	0.04451 (9)	0.0161 (2)	
C20	0.42361 (17)	0.65689 (14)	−0.06757 (9)	0.0173 (2)	
H20A	0.373241	0.760065	−0.111895	0.021*	
C21	0.49260 (16)	0.49242 (13)	0.10978 (9)	0.0161 (2)	
C10	0.78669 (18)	0.91129 (15)	0.42350 (10)	0.0203 (2)	
H10	0.893561	0.940894	0.448094	0.024*	
C11	0.67585 (19)	1.02447 (15)	0.34729 (11)	0.0239 (3)	
H11	0.707520	1.128836	0.318970	0.029*	
C12	0.51751 (18)	0.98197 (15)	0.31325 (10)	0.0221 (2)	
H12	0.438083	1.057794	0.261594	0.027*	
C22	0.46261 (16)	0.52553 (14)	0.22495 (9)	0.0171 (2)	
C5	0.94801 (16)	0.66816 (14)	0.88063 (9)	0.0168 (2)	
H5	0.903446	0.786937	0.865789	0.020*	
C6	0.96400 (16)	0.58878 (13)	0.98974 (9)	0.0157 (2)	

### N,N'-Bis(pyridin-3-ylmethyl)pyromellitic diimide–naphthalene-2,6-dicarboxylic acid (1/1) Atomic displacement parameters (Å^2^)

**Table:**

	*U*^11^	*U*^22^	*U*^33^	*U*^12^	*U*^13^	*U*^23^
O1	0.0342 (5)	0.0199 (4)	0.0182 (4)	−0.0031 (3)	−0.0112 (3)	−0.0029 (3)
O18	0.0375 (5)	0.0159 (4)	0.0253 (4)	0.0007 (4)	−0.0065 (4)	−0.0037 (3)
O23	0.0314 (5)	0.0212 (4)	0.0178 (4)	−0.0035 (3)	−0.0039 (3)	−0.0003 (3)
O3	0.0315 (5)	0.0200 (4)	0.0205 (4)	−0.0099 (3)	−0.0075 (3)	0.0012 (3)
N9	0.0262 (5)	0.0188 (5)	0.0152 (4)	−0.0048 (4)	−0.0051 (4)	−0.0029 (3)
N16	0.0195 (4)	0.0170 (4)	0.0165 (4)	−0.0040 (3)	−0.0025 (3)	−0.0044 (3)
C7	0.0182 (5)	0.0158 (5)	0.0183 (5)	−0.0031 (4)	−0.0025 (4)	−0.0036 (4)
C8	0.0175 (5)	0.0184 (5)	0.0160 (5)	−0.0041 (4)	−0.0015 (4)	−0.0045 (4)
C4	0.0147 (5)	0.0187 (5)	0.0152 (5)	−0.0047 (4)	−0.0029 (4)	−0.0015 (4)
C2	0.0156 (5)	0.0204 (5)	0.0168 (5)	−0.0048 (4)	−0.0027 (4)	−0.0021 (4)
C14	0.0280 (6)	0.0173 (5)	0.0154 (5)	−0.0077 (4)	−0.0035 (4)	−0.0013 (4)
C13	0.0189 (5)	0.0170 (5)	0.0149 (5)	−0.0033 (4)	−0.0004 (4)	−0.0050 (4)
C15	0.0188 (5)	0.0206 (5)	0.0179 (5)	−0.0047 (4)	−0.0003 (4)	−0.0067 (4)
C17	0.0194 (5)	0.0171 (5)	0.0187 (5)	−0.0043 (4)	−0.0030 (4)	−0.0039 (4)
C19	0.0160 (5)	0.0143 (5)	0.0184 (5)	−0.0041 (4)	−0.0024 (4)	−0.0026 (4)
C20	0.0188 (5)	0.0145 (5)	0.0182 (5)	−0.0038 (4)	−0.0035 (4)	−0.0008 (4)
C21	0.0162 (5)	0.0162 (5)	0.0166 (5)	−0.0053 (4)	−0.0026 (4)	−0.0015 (4)
C10	0.0217 (5)	0.0205 (5)	0.0203 (5)	−0.0062 (4)	−0.0028 (4)	−0.0051 (4)
C11	0.0266 (6)	0.0168 (5)	0.0289 (6)	−0.0074 (4)	−0.0060 (5)	0.0008 (4)
C12	0.0229 (6)	0.0177 (5)	0.0243 (6)	−0.0032 (4)	−0.0059 (4)	0.0007 (4)
C22	0.0169 (5)	0.0167 (5)	0.0183 (5)	−0.0049 (4)	−0.0020 (4)	−0.0032 (4)
C5	0.0173 (5)	0.0161 (5)	0.0171 (5)	−0.0041 (4)	−0.0033 (4)	−0.0014 (4)
C6	0.0141 (5)	0.0167 (5)	0.0167 (5)	−0.0036 (4)	−0.0023 (4)	−0.0030 (4)

### N,N'-Bis(pyridin-3-ylmethyl)pyromellitic diimide–naphthalene-2,6-dicarboxylic acid (1/1) Geometric parameters (Å, º)

**Table:**

O1—C2	1.3135 (14)	C13—C12	1.3900 (16)
O1—H1	0.9501 (10)	C13—C15	1.5080 (15)
O18—C17	1.2073 (14)	C15—H15B	0.9900
O23—C22	1.2094 (14)	C15—H15A	0.9900
O3—C2	1.2182 (14)	C17—C19	1.4921 (15)
N9—C10	1.3335 (15)	C19—C20	1.3867 (15)
N9—C14	1.3391 (15)	C19—C21	1.3924 (15)
N16—C22	1.3911 (14)	C20—C21^ii^	1.3872 (15)
N16—C17	1.3963 (14)	C20—H20A	0.9500
N16—C15	1.4619 (14)	C21—C22	1.4917 (15)
C7—C8	1.3732 (15)	C10—C11	1.3843 (17)
C7—C6^i^	1.4216 (15)	C10—H10	0.9500
C7—H7	0.9500	C11—C12	1.3870 (17)
C8—C4	1.4201 (15)	C11—H11	0.9500
C8—H8	0.9500	C12—H12	0.9500
C4—C5	1.3748 (15)	C5—C6	1.4179 (15)
C4—C2	1.5013 (15)	C5—H5	0.9500
C14—C13	1.3889 (16)	C6—C6^i^	1.422 (2)
C14—H14	0.9500		
			
C2—O1—H1	106.8 (11)	O18—C17—C19	128.73 (11)
C10—N9—C14	118.72 (10)	N16—C17—C19	105.69 (9)
C22—N16—C17	112.33 (9)	C20—C19—C21	122.87 (10)
C22—N16—C15	123.31 (9)	C20—C19—C17	129.04 (10)
C17—N16—C15	124.37 (9)	C21—C19—C17	108.09 (10)
C8—C7—C6^i^	120.49 (10)	C19—C20—C21^ii^	114.62 (10)
C8—C7—H7	119.8	C19—C20—H20A	122.7
C6^i^—C7—H7	119.8	C21^ii^—C20—H20A	122.7
C7—C8—C4	120.09 (10)	C20^ii^—C21—C19	122.52 (10)
C7—C8—H8	120.0	C20^ii^—C21—C22	129.54 (10)
C4—C8—H8	120.0	C19—C21—C22	107.95 (9)
C5—C4—C8	120.49 (10)	N9—C10—C11	122.60 (11)
C5—C4—C2	117.92 (10)	N9—C10—H10	118.7
C8—C4—C2	121.54 (10)	C11—C10—H10	118.7
O3—C2—O1	124.33 (10)	C10—C11—C12	118.46 (11)
O3—C2—C4	121.82 (10)	C10—C11—H11	120.8
O1—C2—C4	113.81 (10)	C12—C11—H11	120.8
N9—C14—C13	122.82 (11)	C11—C12—C13	119.57 (11)
N9—C14—H14	118.6	C11—C12—H12	120.2
C13—C14—H14	118.6	C13—C12—H12	120.2
C14—C13—C12	117.83 (11)	O23—C22—N16	125.12 (10)
C14—C13—C15	121.26 (10)	O23—C22—C21	128.96 (10)
C12—C13—C15	120.91 (10)	N16—C22—C21	105.93 (9)
N16—C15—C13	111.95 (9)	C4—C5—C6	120.56 (10)
N16—C15—H15B	109.2	C4—C5—H5	119.7
C13—C15—H15B	109.2	C6—C5—H5	119.7
N16—C15—H15A	109.2	C5—C6—C7^i^	121.66 (10)
C13—C15—H15A	109.2	C5—C6—C6^i^	118.99 (12)
H15B—C15—H15A	107.9	C7^i^—C6—C6^i^	119.35 (12)
O18—C17—N16	125.59 (11)		
			
C6^i^—C7—C8—C4	1.93 (17)	C17—C19—C20—C21^ii^	−179.30 (11)
C7—C8—C4—C5	−0.73 (17)	C20—C19—C21—C20^ii^	0.13 (19)
C7—C8—C4—C2	−177.93 (10)	C17—C19—C21—C20^ii^	179.46 (10)
C5—C4—C2—O3	18.94 (16)	C20—C19—C21—C22	−179.81 (10)
C8—C4—C2—O3	−163.79 (11)	C17—C19—C21—C22	−0.48 (12)
C5—C4—C2—O1	−158.88 (10)	C14—N9—C10—C11	−1.02 (18)
C8—C4—C2—O1	18.39 (15)	N9—C10—C11—C12	1.23 (19)
C10—N9—C14—C13	0.25 (17)	C10—C11—C12—C13	−0.65 (18)
N9—C14—C13—C12	0.28 (17)	C14—C13—C12—C11	−0.06 (17)
N9—C14—C13—C15	−179.75 (10)	C15—C13—C12—C11	179.98 (11)
C22—N16—C15—C13	83.09 (13)	C17—N16—C22—O23	177.96 (11)
C17—N16—C15—C13	−97.45 (12)	C15—N16—C22—O23	−2.52 (18)
C14—C13—C15—N16	−92.23 (12)	C17—N16—C22—C21	−1.83 (12)
C12—C13—C15—N16	87.73 (13)	C15—N16—C22—C21	177.69 (9)
C22—N16—C17—O18	−178.33 (12)	C20^ii^—C21—C22—O23	1.7 (2)
C15—N16—C17—O18	2.15 (19)	C19—C21—C22—O23	−178.40 (12)
C22—N16—C17—C19	1.54 (12)	C20^ii^—C21—C22—N16	−178.55 (11)
C15—N16—C17—C19	−177.97 (10)	C19—C21—C22—N16	1.39 (12)
O18—C17—C19—C20	−1.4 (2)	C8—C4—C5—C6	−1.07 (16)
N16—C17—C19—C20	178.69 (11)	C2—C4—C5—C6	176.22 (10)
O18—C17—C19—C21	179.28 (12)	C4—C5—C6—C7^i^	−178.81 (10)
N16—C17—C19—C21	−0.59 (12)	C4—C5—C6—C6^i^	1.64 (19)
C21—C19—C20—C21^ii^	−0.12 (18)		

Symmetry codes: (i) −*x*+2, −*y*+1, −*z*+2; (ii) −*x*+1, −*y*+1, −*z*.

### N,N'-Bis(pyridin-3-ylmethyl)pyromellitic diimide–naphthalene-2,6-dicarboxylic acid (1/1) Hydrogen-bond geometry (Å, º)

**Table:**

*D*—H···*A*	*D*—H	H···*A*	*D*···*A*	*D*—H···*A*
O1—H1···N9	0.95 (1)	1.64 (1)	2.5806 (13)	172 (2)
C15—H15*A*···O3^iii^	0.99	2.56	3.4073 (14)	143
C11—H11···O23^iv^	0.95	2.51	3.2402 (15)	134
C10—H10···O3^v^	0.95	2.64	3.2414 (15)	122
C11—H11···O3^v^	0.95	2.62	3.2207 (15)	122

Symmetry codes: (iii) −*x*+1, −*y*+2, −*z*+1; (iv) *x*, *y*+1, *z*; (v) −*x*+2, −*y*+2, −*z*+1.
